# Transforming growth factor-β1 reduces apoptosis via autophagy activation in hepatic stellate cells

**DOI:** 10.3892/mmr.2014.2383

**Published:** 2014-07-15

**Authors:** MEI-YA FU, YA-JUN HE, XIA LV, ZHI-HE LIU, YAN SHEN, GUO-RONG YE, YAN-MEI DENG, JIAN-CHANG SHU

**Affiliations:** Department of Gastroenterology, The Fourth Affiliated Hospital of the Medical College of Jinan University, Guangzhou Red Cross Hospital, Guangzhou, Guangdong 510220, P.R. China

**Keywords:** transforming growth factor-β1, hepatic stellate cell, autophagy, apoptosis

## Abstract

Autophagy is a metabolic process that is important in fibrogenesis, in which cellular components are degraded by lysosomal machinery. Transforming growth factor β1 (TGF-β1) is a potent fibrogenic cytokine involved in liver fibrosis; however, it remains elusive whether autophagy is regulated by TGF-β1 in this process. In the present study, the function of TGF-β1-mediated autophagy in the proliferation and apoptosis of hepatic stellate cells (HSCs) was investigated. A rat HSC cell line (HSC-T6) was incubated with or without TGF-β1 followed by bafilomycin A1, and microtubule-associated proteins 1A/1B light chain 3 (LC3) small interfering (si)RNA was used to inhibit autophagy in order to assess the association between TGF-β1 and autophagy. HSC-T6 cell transient transfection was accomplished with a pLVX-AcGFP-N1-rLC3B-encoding plasmid. An MTS assay and flow cytometry were utilized to detect proliferation and apoptosis of HSC-T6 cells. Quantitative polymerase chain reaction, immunofluorescence and western blot analysis were used to detect the presence of activation markers. Proliferation was increased and apoptosis was reduced in HSC-T6 cells treated with TGF-β1 compared with cells subjected to serum deprivation. However, when HSC-T6 cells were treated with bafilomycin A1 and LC3 siRNA, increased apoptosis and reduced proliferation were observed. In addition, protein and mRNA expression levels of the autophagy marker LC3 were significantly increased. GFP-LC3 punctate markings were more prolific following TGF-β1 treatment of HSC-T6 cells, indicating that TGF-β1 may rescue HSC-T6 cells from serum deprivation and reduce apoptosis via autophagy induction. The present study elucidated the possible functions of TGF-β1-mediated autophagy in the pathological process of liver fibrosis.

## Introduction

Macroautophagy (hereafter referred to as autophagy) is an intracellular catabolic mechanism by which proteins, cellular organelles and invading microbes are degraded by lysosomal machinery (1). Autophagy is associated with aging, autoimmunity, infection, heart disease, cancer and neurodegenerative disorders (2), and shares certain regulatory pathways and molecular mechanisms with apoptosis (3).

While a number of studies examined autophagy in liver disease (4–7), the relationship between autophagy and liver fibrosis has not been broadly investigated (8–10). Hernández-Gea *et al* (11) demonstrated that autophagy releases lipids that promote activated hepatic stellate cells (HSCs) to create fibrotic damage. In addition, Thoen *et al* (12) observed that autophagic flow increased following HSC activation *in vitro*, and that treatment with an autophagy inhibitor partially inhibited HSC activation. While there is support for the theory that autophagy is involved in HSC activation, the underlying molecular mechanisms remain elusive. Autophagy in liver cells may produce distinct physiological and pathological outcomes under different conditions. In the majority of types of liver disorders, including liver ischemia/reperfusion injury and fatty liver disease, and following liver transplantation, autophagy mainly serves a protective role in which it promotes lipid droplet degradation, decreases protein deposition and guards against cell apoptosis (13,14). However, these protective actions of autophagy may activate HSC proliferation by blocking apoptosis, leading to accelerated fibrotic progression (15).

HSC activation and proliferation is an essential mechanism for promoting liver fibrosis (16,17). TGF-β1 is important in HSC activation and proliferation, and TGF-β1 inhibitors have been demonstrated to reduce liver fibrosis in rodents. However, since TGF-β1 is a prototypical cytokine that regulates a plethora of cellular pathways, non-specific inhibition of TGF-β1 is likely to produce multiple unpredictable adverse effects, thereby limiting its application in humans (18).

TGF-β1 induces autophagy activation, which regulates cell proliferation and apoptosis in a cell type-dependent manner (19). In epithelial cells, TGF-β1-induced autophagy inhibited cell growth and promoted apoptosis. However, TGF-β1-induced autophagy inhibited apoptosis in mesenchymal cells (20). In the present study, the effect of TGF-β1 stimulation on autophagy activation in HSC-T6 cells and its regulatory role in HSC-T6 proliferation and apoptosis was investigated.

## Materials and methods

### Cell culture

The activated rat HSC-T6 cell line with SV40 transfection was provided as a gift by Professor Lie-Ming Xu (Division of Liver Diseases, Shanghai University of Traditional Chinese Medicine, Shanghai, China). Cells were cultured in Dulbecco’s modified Eagle’s medium (HyClone, Logan, UT, USA) supplemented with 100 U/ml penicillin, 100 U/ml streptomycin, and 10% fetal bovine serum (FBS; HyClone). Cells were incubated at 37°C with 5% CO_2_ in a humidified incubator and medium was replaced every two days.

### Plasmid and cell transfection

HSC-T6 cells were seeded in six-well plates (2×10^5^/well) and transfected with PLVX-AcGFP-N1-LC3B (Shanghai ExCell Biology, Inc., Shanghai, China) or vector alone. The cells were used for subsequent experiments 48 h post transfection.

### Cell proliferation assay

Cell viability was measured using the 3-(4,5-dimethylthiazol-2-yl)-5-(3-carboxymethoxyphenyl)-2-(4-sulfophenyl)-2H-tetrazolium (MTS) assay (Promega, Madison, WI, USA). Cells were plated at a density of 1.0×10^5^/well in 96-well plates and incubated with MTS for 4 h at 37°C with 5% CO_2_ in a humidified incubator. Absorbance at 570 nm was measured on a SpectraMax Plus384 microplate reader (Molecular Devices, Sunnyvale, CA, USA).

### Western blot analysis

Protein concentrations were determined using the bicinchoninic acid (BCA) Protein Assay kit (Pierce, Rockford, IL, USA). Samples of 30 μg total protein were used for western blots. Primary antibodies were as follows: Rabbit polyclonal anti-LC3B (1/1,000; Cell Signaling, Danvers, MA, USA), rabbit polyclonal anti-GAPDH (1/1,000) and rabbit polyclonal anti-cleaved caspase-3 (1/1,000) (Shanghai ExCell Biology, Inc.). Goat anti-rabbit immunoglobulin (Ig) G-horseradish peroxidase (1/10,000; Shanghai ExCell Biology, Inc.) was used as the secondary antibody for detection. Protein bands were visualized using SuperSignal West Pico Chemiluminescent Substrate (Thermo Fisher Scientific, Waltham, MA, USA).

### RNA isolation and quantitative polymerase chain reaction (qPCR)

Total RNA from cultured cells was extracted using TRIzol reagent (Invitrogen Life Technologies, Carlsbad, CA, USA). RNA was reverse-transcribed using SuperScript II Reverse Transcriptase (Invitrogen, Life Technologies) at 65°C for 5 min, 42°C for 50 min, and 70°C for 15 min. Gene-specific primers (MAP1LC3) used in the present study were purchased from Invitrogen and had the following sequences: Map1LC3, reverse ACCAAGCCTTCTTCCTCC and forward GCTCTTCTATTTCAAGTCCCTA; GADPH, reverse ATGGTGGTGAAGACGGTA and forward GGCACAGTCAAGGCTGAGAATG. Maxima^®^ SYBR Green qPCR Master Mix (Thermo Fisher Scientific, Waltham, MA, USA), cDNA template, and primer were mixed at a final volume of 20 μl and subjected to qPCR in an ABI 7500 Real-Time PCR system (Applied Biosystems, Foster City, CA, USA). Data were analyzed using the ΔΔ threshold (Ct) method, and Ct values were normalized to GAPDH, which served as an internal control.

### Flow cytometric analysis of apoptosis

HSC-T6 cells were seeded in 12-well plates (0.5×10^5^/well) and incubated at 37°C with 5% CO_2_. After 24 h, medium was removed and serum-free Hank’s balanced salt solution (137.93 mM NaCl, 5.33 mM KCl, 4.17 mM NaHCO_3_, 0.441 mM KH_2_PO_4_, 0.338 mM Na_2_HPO_4_, 5.56 mM D-Glucose) was added. Cells were then treated with 10 ng/ml TGF-β1 (PeproTech, Rocky Hill, NJ, USA). Following TGF-β1 treatment, cells were harvested, washed in cold phosphate-buffered saline, double-stained with fluorescein isothiocyanate (FITC)-conjugated Annexin V and propidium iodide (Merck, Darmstadt, Germany) and analyzed by flow cytometry (BD Biosciences, San Jose, CA, USA).

### Immunofluorescence

HSC-T6 cells were seeded in six-well plates (2×10^5^/well) on cover slips. Cells were formalin-fixed for 30 min, immersed in 0.2% Triton X-100 and rabbit polyclonal anti-LC3B for 10 min and incubated with FITC-conjugated AffiniPure Goat Anti-rabbit IgG (1/50) and DAPI (Shanghai ExCell Biology, Inc.). Cells were subsequently observed under a fluorescence microscope (LSM 510 META, Zeiss, Jena, Germany).

### Statistical analysis

All experiments were repeated a minimum of three times. All data are expressed as the mean ± standard error. Differences between groups were assessed using one-way analysis of variance or independent sample t-test. All statistical analyses were performed using SPSS, version 17.0 (SPSS, Inc., Chicago, IL, USA), and P<0.05 was considered to represent a statistically significant difference.

## Results

### TGF-β1 ameliorates HSC-T6 apoptosis induced by serum deprivation

**Figure 1:**
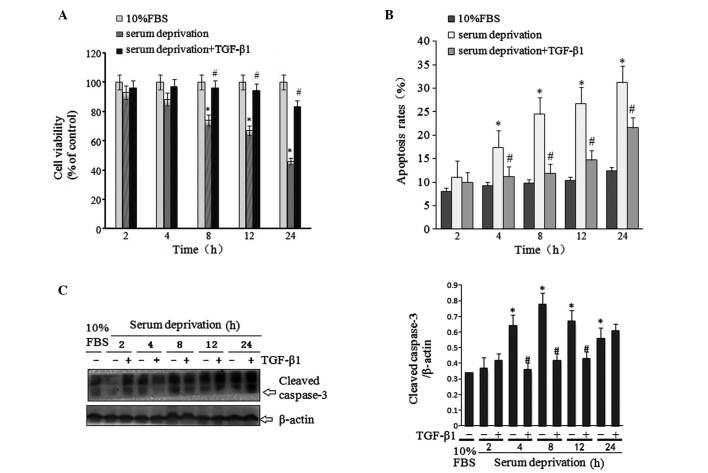
TGF-β1 ameliorates HSC-T6 cell apoptosis induced by serum deprivation. (A) Cell viability assessed by MTS assay; (B) western blot analysis of caspase-3 protein expression in cells with or without TGF-β1 treatment; and (C) cell apoptosis levels assessed by flow cytometry following incubation with 10% FBS, serum-deprived medium or serum-deprived medium with TGF-β1 (10 ng/ml) treatment for 6 h. ^*^P<0.05 vs. control (10% FBS); ^#^P<0.05 vs. serum deprivation without TGF-β1 treatment. FBS, fetal bovine serum; TGF-β1, transforming growth factor β1; MTS, 3-(4,5-dimethylthiazol-2-yl)-5-(3-carboxymethoxyphenyl)-2-(4-sulfophenyl)-2H-tetrazolium.

The MTS assay was utilized to determine the viability of HSC-T6 cells under various treatment conditions. The viability of cells in medium containing 10% FBS was stable for the 24-h experimental period, while the viability of cells in serum-free medium was significantly reduced in a time-dependent manner. Of note, treatment with TGF-β1 (10 ng/ml) significantly ameliorated this serum deprivation-induced reduction in cell viability (Fig. 1A). Western blots were then conducted to determine protein expression levels of caspase-3, a key regulator of apoptosis. The results indicated that TGF-β1 treatment significantly reduced serum deprivation-induced caspase-3 expression (Fig. 1B), in line with the effect of TGF-β1 on cell viability. In addition, flow cytometry was used to study the effect of TGF-β1 on cell apoptosis. The results indicated that serum deprivation induced significant cell apoptosis, which was ameliorated by TGF-β1 treatment (Fig. 1C).

### TGF-β1 induces mRNA expression of the autophagic marker Map1LC3

**Figure 2:**
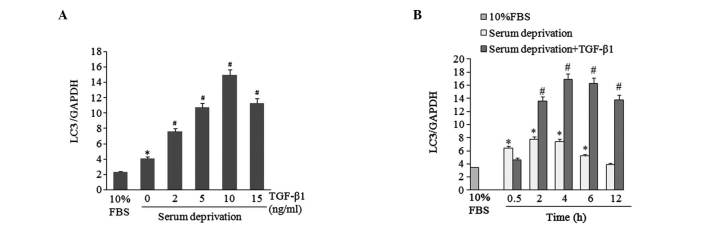
TGF-β1 increases Map1LC3 mRNA expression levels in HSC-T6 cells. (A) Cells were cultured in 10% FBS or serum-free medium with different concentrations of TGF-β1 for 4 h. Total RNA was isolated and LC3 mRNA was measured following amplification by quantitative polymerase chain reaction; (B) Cells were cultured in serum-free medium with or without TGF-β1 (10 ng/ml) for various time periods. ^*^P<0.05 vs. control (10% FBS); ^#^P<0.05 vs. serum deprivation without TGF-β1 treatment. FBS, fetal bovine serum; TGF-β1, transforming growth factor β1; Map1LC3, microtubule-associated proteins 1A/1B light chain 3.

The levels of LC3II, a proteolytic product of the Map1LC3 protein, is closely associated with the number of autophagic lysosomes present, and is widely used as a molecular marker for autophagy (21). In the present study, mRNA expression levels of Map1LC3 in HSC-T6 cells were analyzed under various treatment conditions. HSC-T6 cells were incubated in 10% FBS or serum-free medium with different concentrations of TGF-β1 (0, 2, 5, 10, 15 ng/ml) for 4 h. qPCR results indicated that serum deprivation induced Map1LC3 mRNA expression in HSC-T6 cells, and TGF-β1 treatment further elevated the levels in a dose-dependent manner (Fig. 2A). LC3 mRNA expression levels were then determined in HSC-T6 cells treated with 10 ng/ml TGF-β1 for various time periods. From 2 to 12 h, TGF-β1 stimulated higher levels of LC3 mRNA expression than those stimulated by serum deprivation alone (Fig. 2B).

### TGF-β1 increases LC3II protein levels

**Figure 3:**
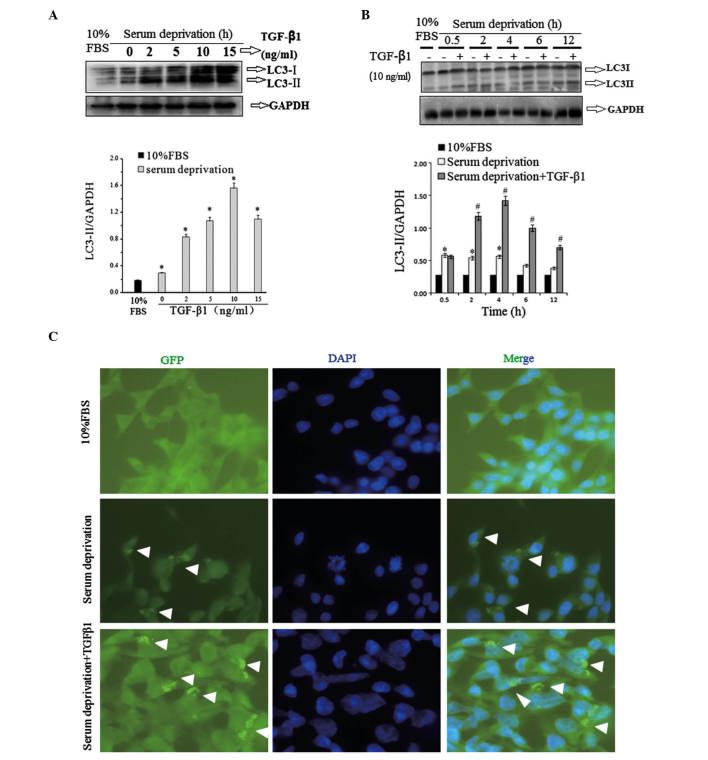
TGF-β1 increases LC3II protein levels in HSC-T6 cells. (A) Cells were cultured in 10% FBS or serum-free medium with different concentrations of TGF-β1 for 4 h. ^*^P<0.05 vs. control (10% FBS). (B) Cells were cultured in 10% FBS or serum-free medium with or without TGF-β1 (10 ng/ml) for various time periods. ^*^P<0.05 vs. control (10% FBS); ^#^P<0.05 vs. serum deprivation without TGF-β1 treatment; (C) TGF-β1 induces autophagic punctate formation (scale bar=20 μm, arrow). HSC-T6 cells were transiently transfected with GFP-LC3 for 48 h. Immunofluorescence staining was performed to detect LC3 aggregation by fluorescence microscopy in cells incubated in 10% FBS or serum-free medium with or without TGF-β1 for 6 h. FBS, fetal bovine serum; TGF-β1, transforming growth factor β1; GFP, green fluorescent protein; LC3, microtubule-associated proteins 1A/1B light chain 3.

Levels of LC3II protein were also measured by western blot analysis to enable further consideration of the effect of TGF-β1 on autophagy in HSC-T6 cells. The cells subjected to western blotting were treated under similar conditions to those used in the Map1LC3 mRNA expression analysis. 4-h serum deprivation significantly increased LC3II protein levels in HSC-T6 cells compared with levels in FBS-cultured cells, and TGF-β1 treatment further elevated the LC3II protein levels in a dose-dependent manner (Fig. 3A). In the time-course study, it was observed that from 2 to 12 h, TGF-β1 (10 ng/ml) treatment increased LC3II protein levels to a greater extent than serum deprivation alone (Fig. 3B). The results indicated that the LC3II protein levels were associated with Map1LC3 mRNA expression.

### Detection of GFP-LC3 fusion protein aggregation in HSC-T6 cells by immunofluorescence staining

Immunofluorescence staining exhibited a clear increase in GFP-LC3 autophagic punctate in the TGF-β1-treated group compared with the group with serum deprivation without TGF-β1 (Fig. 3C). Autophagic punctate was calculated using image-pro-plus 5.0 software analysis (Media Cybernetics, Bethesda, MD, USA). GFP-LC3 aggregation in cells with serum deprivation (11.70±1.31, P>0.05 vs. 10% FBS group) and cells with serum deprivation combined with TGF-β1 treatment (19.33±2.76, P<0.05 vs. 10% FBS group) was greater than the level of aggregation in the control group (7.32±0.85).

### TGF-β1 inhibits HSC-T6 cell apoptosis through induction of autophagy

**Figure 4:**
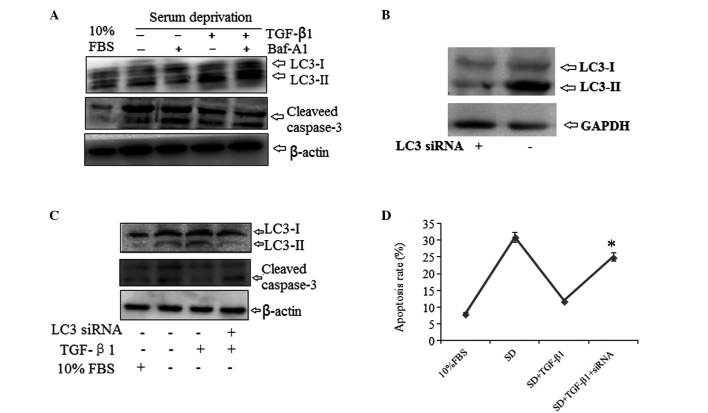
TGF-β1 inhibits HSC-T6 cell apoptosis through induction of autophagy. (A) Total cell lysates from cells incubated with (+) or without (−) TGF-β1 (10 ng/ml) and Baf-A1 (100 nM) for 12 h; (B) Cells transfected with specific LC3 siRNA (+) or control siRNA (−) and incubated in serum-free medium with (+) or without (−) TGF-β1 (10 ng/ml); (C) Cells incubated in medium with (+) or without (−) TGF-β1 (10 ng/ml) and transient transfection with LC3 siRNA for 48 h; (D) Flow cytometry indicating apoptotic rate of cells. ^*^P<0.05 vs. SD + TGF-β1. FBS, fetal bovine serum; TGF-β1, transforming growth factor-β1; BAF-A1, bafilomycin A1; siRNA, small interfering RNA; SD, serum deprivation; LC3, microtubule-associated proteins 1A/1B light chain 3.

To evaluate the role of autophagy in the anti-apoptotic effect of TGF-β1 in serum-deprived HSC-T6 cells, the effect of the specific autophagy inhibitor bafilomycin A1 and the effect of LC3 gene knockdown were assessed. Bafilomycin A1 treatment significantly reduced the inhibitory effect of TGF-β1 on cleaved caspase-3 expression (Fig. 4A). In addition, HSC-T6 cells were transfected with a specific LC3 small interfering (si)RNA for 24–48 h in order to knockdown the LC3 gene (Fig. 4B). When serum-deprived cells were treated with TGF-β1, cells transfected with LC3 siRNA displayed visibly increased cleaved caspase-3 levels compared with cells transfected with the vector without TGF-β1 treatment (Fig. 4C). The effect of LC3 knockdown on cell apoptosis was also evaluated using flow cytometry. The anti-apoptotic effect of TGF-β1 in serum-deprived HSC-T6 cells was significantly reduced in cells transfected with LC3 siRNA (Fig. 4D). These results implied that TGF-β1 acted through autophagy activation to ameliorate serum deprivation-induced apoptosis in HSC-T6 cells.

## Discussion

In the present study, serum deprivation was noted to significantly increase levels of apoptosis in HSC-T6 cells, accompanied by autophagy activation (measured by LC3II levels and protein aggregation). It was also demonstrated that TGF-β1 ameliorated serum deprivation-induced apoptosis via activation of autophagy.

TGF-β1, which is one of the strongest inducers of extracellular matrix (ECM) production, serves a central purpose in the process of fibrogenesis in fibrotic diseases (22,23). TGF-β1 exhibits pro-apoptotic or anti-apoptotic effects depending on the cell type, in addition to other factors (24,25). The pro-apoptotic effects of TGF-β1 are well documented in various cell types, including immune cells (26) and epithelial cells (27). However, TGF-β1 has also been demonstrated to inhibit apoptosis in certain cell types, including murine macrophages (28), mesenchymal cells (29), fibroblasts and myofibroblasts (30). In the present study, TGF-β1 was demonstrated to protect HSC-T6 cells against the apoptosis and total cell death caused by serum deprivation.

Multiple previous studies have demonstrated the central role of TGF-β1 in the initiation and development of liver fibrosis: One study indicated that TGF-β1 simultaneously induces autophagy and apoptosis in mammary epithelial cells (19). However, in mesangial cells, TGF-β1-induced autophagy has been demonstrated to produce anti-apoptotic effects (20). Upon activation of autophagy, the cytoplasmic protein form of LC3 (LC3I) is processed and recruited to autophagosomes, where LC3II is generated by site-specific proteolysis and lipidation near the C-terminus. Thus, the formation of cellular autophagosome punctate containing LC3II is a marker of autophagic activation. The results of the current study indicated that TGF-β1 treatment increased LC3 mRNA expression in serum-deprived HSC-T6 cells in a concentration- and time-dependent manner. In addition, TGF-β1 increased LC3II protein levels in a similar manner.

Autophagy can be monitored by GFP-LC3 labeling, in which formation of autophagosome punctate containing GFP-LC3 is detected by indirect immunofluorescence staining or direct fluorescence microscopy (21). In the present study, this method was used to observe that in HSC-T6 cells transiently transfected with GFP-LC3, LC3 punctate aggregated around the nuclear membrane. TGF-β1 treatment led to the relocation of LC3 from the cytoplasm to the caryotheca, indicating that TGF-β1 induces autophagy in HSC-T6 cells.

Autophagy is closely associated with apoptosis and shares similar signaling pathways and stimulating factors, differing only in the threshold values (3). In general, the relationship between autophagy and apoptosis is mutual inhibition. Autophagy activation may inhibit cell apoptosis, but excessive autophagy may cause autophagic cell death (31). TGF-β1 is an important regulator of autophagy and apoptosis (32,33), and in the present study, it was demonstrated that in serum-deprived cells, incubation with TGF-β1 significantly suppressed cleaved caspase-3 expression levels. Blocking autophagy activation with bafilomycin A1 or LC3 siRNA significantly reduced the inhibitory effect of TGF-β1 on cleaved caspase-3 expression. TGF-β1 regulates autophagy through both SMAD and non-SMAD signaling pathways (33); however, the precise mechanism by which TGF-β1 regulates autophagy in HSC-T6 cells requires further research.

In conclusion, the present study demonstrated that TGF-β1 ameliorated apoptosis in serum-deprived HSC-T6 cells via a mechanism mediated by autophagy activation. Further studies are required to elucidate the mechanism by which TGF-β1 activates autophagy in HSC-T6 cells *in vitro* and *in vivo*.

## References

[1] Ni HM, Williams JA, Yang H, Shi YH, Fan J, Ding WX (2012). Targeting autophagy for the treatment of liver diseases. Pharmacol Res.

[2] Klionsky DJ (2010). The autophagy connection. Dev Cell.

[3] González-Polo RA, Boya P, Pauleau AL (2005). The apoptosis/autophagy paradox: autophagic vacuolization before apoptotic death. J Cell Sci.

[4] Komatsu M (2012). Liver autophagy: physiology and pathology. J Bio Chem.

[5] Sasaki M, Miyakoshi M, Sato Y, Nakanuma Y (2012). Autophagy may precede cellular senescence of bile ductular cells in ductular reaction in primary biliary cirrhosis. Dig Dis Sci.

[6] Bhogal RH, Afford SC, Bailly Yannick (2013). Autophagy and the Liver. Autophagy - A Double-Edged Sword - Cell Survival or Death?.

[7] Rautou PE, Mansouri A, Lebrec D, Durand F, Valla D, Moreau R (2010). Autophagy in liver diseases. J Hepatol.

[8] Hilscher M, Hernandez-Gea V, Friedman SL (1831). Autophagy and mesenchymal cell fibrogenesis. Biochim Biophys Acta.

[9] Hidvegi T, Ewing M, Hale P (2010). An autophagy-enhancing drug promotes degradation of mutant alpha1-antitrypsin Z and reduces hepatic fibrosis. Science.

[10] Kim SI, Na HJ, Ding Y, Wang Z, Lee SJ, Choi ME (2012). Autophagy promotes intracellular degradation of type I collagen induced by transforming growth factor (TGF)-β1. J Biol Chem.

[11] Hernández-Gea V, Ghiassi-Nejad Z, Rozenfeld R (2012). Autophagy releases lipid that promotes fibrogenesis by activated hepatic stellate cells in mice and in human tissues. Gastroenterology.

[12] Thoen LF, Guimarães EL, Dollé L (2011). A role for autophagy during hepatic stellate cell activation. J Hepatol.

[13] Yin XM, Ding WX, Gao W (2008). Autophagy in the liver. Hepatology.

[14] Rubinsztein DC, Gestwicki JE, Murphy LO, Klionsky DJ (2007). Potential therapeutic applications of autophagy. Nat Rev Drug Discov.

[15] Deretic V, Levine B (2009). Autophagy, immunity and microbial adaptations. Cell Host Microbe.

[16] Friedman SL (2008). Hepatic fibrosis - overview. Toxicology.

[17] Brenner DA (2009). Molecular pathogenesis of liver fibrosis. Trans Am Clin Climatol Assoc.

[18] Iimuro Y, Brenner DA (2008). Matrix metalloproteinase gene delivery for liver fibrosis. Pharm Res.

[19] Gajewska M, Gajkowska B, Motyl T (2005). Apoptosis and autophagy induced by TGF-β1 in bovine mammary epithelial BME-UV1 cells. J Physiol Pharmacol.

[20] Ding Y, Kim JK, Kim SI, Na HJ, Jun SY, Lee SJ, Choi ME (2010). TGF-{beta}1 protects against mesangial cell apoptosis via induction of autophagy. J Biol Chem.

[21] Klionsky DJ, Abdalla FC, Abeliovich H (2012). Guidelines for the use and interpretation of assays for monitoring autophagy. Autophagy.

[22] Leask A, Abraham DJ (2004). TGF beta signaling and the fibrotic response. FASEB J.

[23] Nakerakanti S, Trojanowska M (2012). The role of TGF-β receptors in fibrosis. Open Rheumatol J.

[24] Roberts AB (1998). Molecular and cell biology of TGF-beta. Miner Electrolyte Metab.

[25] Roberts AB, Sporn MB (1993). Physiological actions and clinical applications of transforming growth factor-beta (TGF-beta). Growth Factors.

[26] Brown TL, Patil S, Cianci CD, Morrow JS, Howe PH (1999). Transforming growth factor-beta induces caspase 3-independent cleavage of alphaII-spectrin (alpha-fodrin) coincident with apoptosis. J Biol Chem.

[27] Dai C, Yang J, Liu Y (2003). Transforming growth factor-beta1 potentiates renal tubular epithelial cell death by a mechanism independent of Smad signaling. J Biol Chem.

[28] Chin BY, Petrache I, Choi AM, Choi ME (1999). Transforming growth factor beta1 rescues serum deprivation-induced apoptosis via the mitogen-activated protein kinase (MAPK) pathway in macrophages. J Biol Chem.

[29] Hara T, Kamura T, Nakayama K, Oshikawa K, Hatakeyama S, Nakayama K (2001). Degradation of p27(Kip1) at the G(0)–G(1) transition mediated by a Skp2-independent ubiquitination pathway. J Biol Chem.

[30] Chen HH, Zhao S, Song JG (2003). TGF-beta1 suppresses apoptosis via differential regulation of MAP kinases and ceramide production. Cell Death Differ.

[31] Maiuri MC, Zalckvar E, Kimchi A, Kroemer G (2007). Self-eating and self-killing: crosstalk between autophagy and apoptosis. Nat Rev Mol Cell Biol.

[32] Wang RC, Levine B (2010). Autophagy in cellular growth control. FEBS Lett.

[33] Suzuki HI, Kiyono K, Miyazono K (2010). Regulation of autophagy by transforming growth factor-β (TGF-β) signaling. Autophagy.

